# Strongyle-Type Gastrointestinal Nematode Egg Positivity, Fecal Egg Counts, and Associated Factors in Sheep from Guanujo Parish, Ecuador

**DOI:** 10.3390/vetsci13090934

**Published:** 2026-09-09

**Authors:** María Felicidad Morejón-García, Favian Bayas-Morejón

**Affiliations:** Programa de Maestría en Ciencias Veterinarias, Departamento de Posgrado y Educación Continua, Facultad de Ciencias Agropecuarias, Recursos Naturales y del Ambiente, Universidad Estatal de Bolívar, Guaranda 020150, Ecuador; mmorejon@ueb.edu.ec

**Keywords:** sheep, strongyle-type eggs, gastrointestinal nematodes, fecal egg count, modified McMaster technique, stocking density, Ecuador

## Abstract

Background: Strongyle-type gastrointestinal nematodes are among the most important parasitic infections affecting sheep production because of the health and economic losses they cause. Aim: The aim of this study was to determine the positivity, parasite burden, and factors associated with positivity to strongyle-type gastrointestinal nematode eggs in sheep from Guanujo parish, Ecuador. Methods: A quantitative, descriptive, cross-sectional study was conducted between November 2025 and March 2026. Seventy fecal samples were collected individually via rectal sampling from sheep randomly selected within 14 participating farms. The samples were analyzed using coproparasitological techniques with the modified McMaster method to quantify eggs per gram of feces (EPG). Subsequently, univariate analysis and multivariable logistic regression were performed to identify factors associated with positivity for strongyle-type gastrointestinal nematode eggs. Results: The overall positivity was 54.3% (38/70), with a mean parasite burden of 1150 EPG. Mild and moderate infections predominated, although severe infections were also detected. After multivariable adjustment, younger age was consistently associated with positivity. High stocking density was associated with positivity in the primary model, although this association was less stable in the mixed-effects sensitivity analysis. Sheep younger than 12 months showed higher odds of positivity than adults (adjusted OR = 8.75; 95% CI: 3.42–22.38; *p* < 0.001), while animals from farms with high stocking density also showed higher odds of positivity (adjusted OR = 2.69; 95% CI: 1.21–5.97; *p* = 0.015). Conclusions: Younger age was the most consistent factor associated with strongyle-type gastrointestinal nematode egg positivity. High stocking density showed higher odds of positivity in the primary analysis, but this association was not statistically significant in the mixed-effects sensitivity analysis and should therefore be interpreted cautiously.

## Simple Summary

Intestinal parasites pose a significant problem for sheep farming, affecting animal health and potentially causing economic losses. This study evaluated the presence, intensity, and main factors related to infection in 70 sheep from 14 farms in the Guanujo parish, Ecuador. The results showed that 54.3% of the animals were positive for strongyle-type gastrointestinal nematode eggs, primarily at mild to moderate levels, although some severe cases were also identified. Sheep younger than 12 months showed consistently higher odds of strongyle-type egg positivity. High stocking density was associated with positivity in the primary analysis, although this association was less stable in the sensitivity analysis. Strongyle-type egg positivity was frequently detected among the sampled sheep and participating farms. These results will inform prevention and control measures, improve animal health, and promote more sustainable sheep production.

## 1. Introduction

Gastrointestinal parasitosis is a major health and production constraint affecting sheep worldwide, particularly under extensive and semi-intensive production systems. Among the most relevant parasites are strongyle-type gastrointestinal nematodes, which include several genera of veterinary importance and are associated with reduced weight gain, impaired feed efficiency, decreased productivity, and, in severe infections, increased morbidity and mortality [1,2,3,4].

Strongyle-type gastrointestinal nematodes include several genera of veterinary importance, including *Haemonchus*, *Trichostrongylus*, *Teladorsagia*, *Ostertagia*, *Cooperia*, and *Nematodirus*. These parasites are among the main agents affecting grazing sheep due to their negative effects on health, growth, and productivity, as well as the economic losses they generate in small ruminant production systems [3]. Among them, *Haemonchus* and *Trichostrongylus* are among the genera most frequently reported in sheep and goats worldwide, although their distribution, prevalence, and pathogenicity can vary depending on environmental conditions, host susceptibility, and management practices [3,4].

The clinical manifestations associated with these nematodes depend on the genus involved, the parasite load, the age, immune status, and nutritional condition of the host. Moderate to severe infections can cause anemia, enteritis, diarrhea, decreased body condition and weight gain, reduced productivity, and, in severe cases, mortality, especially in young or immunocompromised animals [4]. Although *Haemonchus contortus* is a particularly pathogenic species due to its hematophagous behavior and high reproductive capacity [5,6,7], the eggs of this and other trichostrongylids have similar morphological characteristics, so their presence cannot be specifically attributed to using coproparasitological techniques based solely on egg morphology.

Strongyle-type gastrointestinal nematodes have a direct life cycle, with free-living environmental stages (egg, L1, L2, and infective L3 larvae) and parasitic stages within the host gastrointestinal tract. Temperature and humidity significantly influence the development and survival of the environmental stages; infection occurs primarily when sheep ingest infective L3 larvae while grazing [2,8,9]. These characteristics favor the persistence of transmission in grazing systems when suitable environmental conditions and fecal contamination of pastures are present [3].

The occurrence and burden of strongyle-type gastrointestinal nematodes are determined by the interaction of environmental, individual, and management factors. Among the main factors described are climatic conditions, age, physiological state, production system, animal density, grazing management, and sanitary practices [10,11]. Warm and humid environments favor the development and survival of infective larvae in pastures, while various epidemiological studies have reported higher positivity rates in young animals, herds with irregular deworming programs, and animals with low body condition; however, these associations can vary according to local environmental and production conditions [12,13,14].

Control of strongyle-type gastrointestinal nematodes is further complicated by the increasing occurrence of anthelmintic resistance, which has reduced the effectiveness of conventional treatment programs in different sheep-production systems [15,16]. Consequently, integrated parasite-control approaches combining appropriate anthelmintic use, fecal egg-count monitoring, grazing management, and other preventive measures have been increasingly recommended [17,18,19,20]. Nevertheless, the design of locally appropriate control strategies requires epidemiological information on parasite occurrence, fecal egg counts, and the animal- and farm-level characteristics associated with positivity.

In Ecuador, sheep production is concentrated mainly in the Andean region, including Bolívar Province, where extensive and semi-intensive production systems and traditional management practices are common. Previous studies in Ecuador have documented gastrointestinal nematode infections in sheep [19]; however, local epidemiological information remains limited, particularly regarding strongyle-type gastrointestinal nematode egg positivity and its association with animal and farm-management characteristics in Guanujo parish. This lack of information limits the establishment of baseline epidemiological data for this production area.

Importantly, although several strongyle-type gastrointestinal nematode genera of veterinary importance may infect sheep, their eggs are morphologically similar, and the modified McMaster technique used in the present study does not permit reliable genus- or species-level differentiation. Therefore, the diagnostic outcome of this study was defined as positivity to strongyle-type gastrointestinal nematode eggs, and no genus- or species-specific attribution was made.

Therefore, the aim of this study was to determine the positivity, fecal egg counts, and factors statistically associated with strongyle-type gastrointestinal nematode egg positivity in sheep from participating farms in Guanujo parish, Bolívar Province, Ecuador. The study was intended to generate baseline epidemiological information for the sampled sheep and farms and to support future, larger-scale investigations and integrated parasite-management strategies under Andean sheep-production conditions.

## 2. Materials and Methods

### 2.1. Study Area and Study Period

In this study, sheep fecal samples were collected in the parish of Guanujo and parasitologically analyzed at the laboratories of the Faculty of Agricultural Sciences of the Universidad Estatal de Bolívar between November 2025 and March 2026.

### 2.2. Methodological Approach and Study Design

The study employed a quantitative, observational, cross-sectional, descriptive, and analytical design. During the defined study period, fecal samples and animal- and farm-level information were collected to estimate strongyle-type gastrointestinal nematode egg positivity and fecal egg counts and to evaluate factors statistically associated with positivity.

### 2.3. Study Population, Unit of Analysis, and Unit of Observation

#### 2.3.1. Study Population

In the investigation, the population consisted of approximately 650 sheep belonging to production units in the Guanujo parish.

#### 2.3.2. Unit of Analysis and Unit of Observation

The unit of analysis and observation was each sampled sheep. An individual fecal sample was collected from every animal, together with animal-level and farm-level information on productive, sanitary, and management characteristics.

### 2.4. Sample Size Calculation

Based on an estimated population of 650 sheep in the parish of Guanujo, the minimum sample size was calculated using the formula for estimating a proportion in a finite population. A 95% confidence level (Z = 1.96), an expected positivity of 50% (*p* = 0.50) due to the lack of previous studies in the area, and an absolute margin of error of 10% (d = 0.10) were used. The minimum sample size was 60 animals. To compensate for potential sample losses or unsuitable samples, 70 sheep were ultimately included. The sample size was considered adequate for estimating positivity but may have limited the precision of multivariable analyses performed at the farm level.

### 2.5. Farm Selection and Sampling Procedure

**Figure 1:**
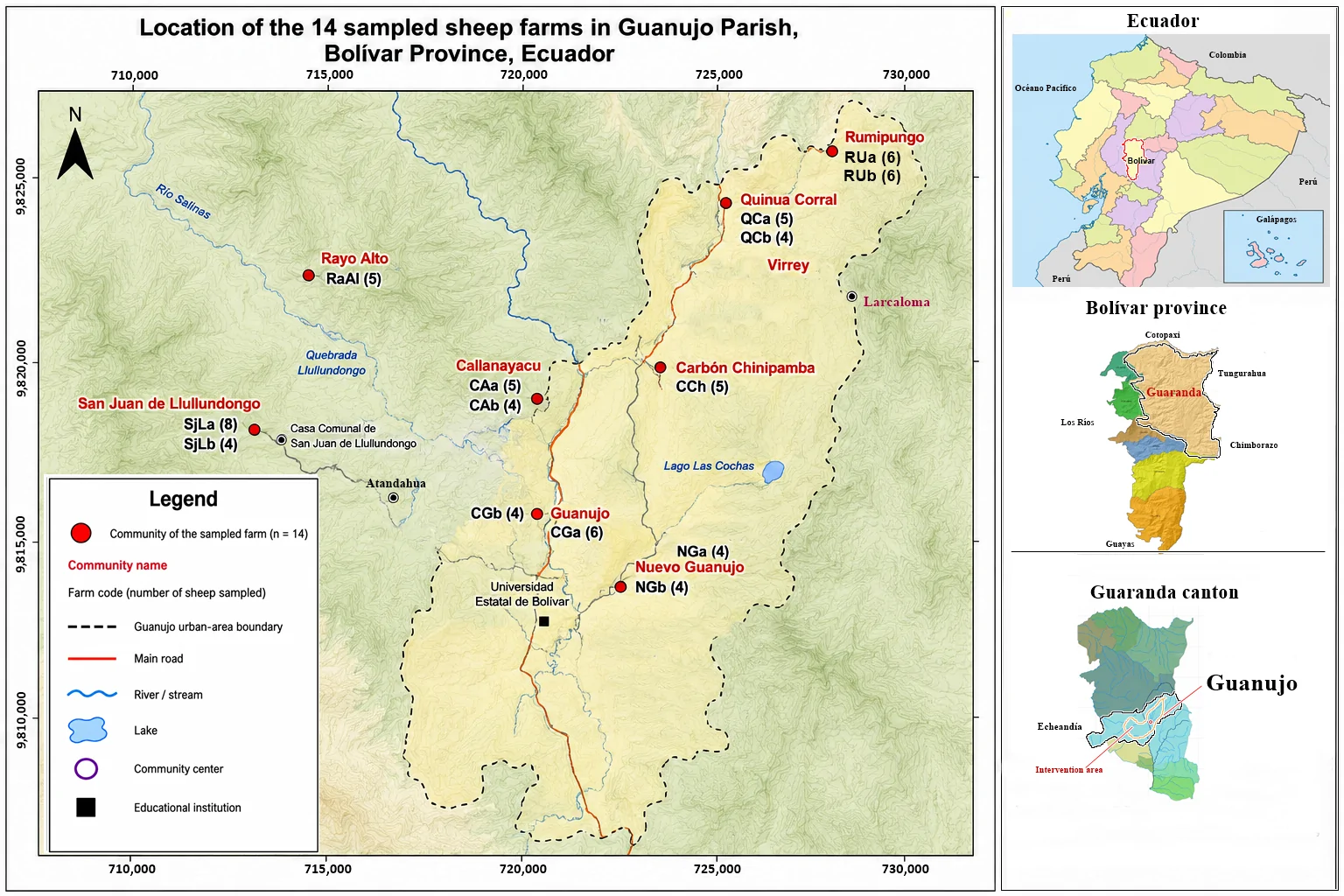
Geographic location of the 14 sheep farms included in the study in Guanujo Parish, Bolívar Province, Ecuador.

Fourteen sheep farms located in the parish of Guanujo were selected using convenience sampling, considering accessibility and the owners’ willingness to participate. The geographical distribution of the participating farms within the study area is shown in Figure 1, while the number of sheep sampled from each farm is provided in Table S1.

At each farm, sequential identification codes and numbers were assigned to the sheep, and individual animals were selected using simple random sampling with a random number procedure generated in Microsoft Excel until the predetermined number of animals for each farm was reached.

### 2.6. Study Population and Animal Classification

All sheep included in the study were crossbred Merino × East Friesian, a genetic composition commonly found among the participating farms in the study area. Animals were classified according to age as lambs (<6 months), juveniles (6–12 months), or adults (>12 months). Physiological status was categorized as suckling, pregnant, non-pregnant, or growing. This population composition allowed the evaluation of strongyle-type gastrointestinal nematode egg positivity across different age and physiological categories, facilitating the assessment of these variables as potential factors statistically associated with positivity.

### 2.7. Farm Management Characteristics

Stocking density was calculated at the farm level as the total number of sheep divided by the effective grazing area, expressed as sheep per hectare (sheep/ha). Based on the observed distribution of stocking densities across the participating farms and the natural separation between categories, farms were classified as having low–medium stocking density (<20 sheep/ha) or high stocking density (≥20 sheep/ha). Stocking densities ranged from 8.0 to 12.5 sheep/ha among farms classified as low–medium and from 20.0 to 50.0 sheep/ha among farms classified as high (Table S3).

The 20 sheep/ha cut-off was used as an operational threshold based on the observed distribution of the participating farms and should not be interpreted as a universally established epidemiological threshold.

### 2.8. Fecal Sample Collection, Preservation, and Transport

Approximately 10–20 g of feces was collected directly from the rectum of each sheep using disposable gloves. Samples were placed in sterile, airtight, individually labeled containers and transported under refrigerated conditions at approximately 4 °C. Coproparasitological analyses were performed within four hours after collection.

### 2.9. Coproparasitological Analysis

All fecal samples were analyzed using the modified McMaster technique to determine the presence and fecal egg counts of strongyle-type gastrointestinal nematode eggs. Briefly, 4 g of feces were homogenized with a flotation solution to obtain the appropriate dilution for egg counting. The resulting suspension was filtered and introduced into a 0.15 mL McMaster counting chamber, following the standard operating procedure for the technique. The egg count was multiplied by a conversion factor of 50, and the results were expressed as eggs per gram of feces (EPG). Eggs compatible with strongyle-type gastrointestinal nematodes were identified based on their morphological characteristics observed using light microscopy, including their oval shape, thin, transparent shell, and the presence of a morula composed of multiple blastomeres, following standard parasitological identification keys. Because eggs of trichostrongylid nematodes are morphologically indistinguishable by fecal examination, parasite identification was limited to strongyle-type eggs and did not permit genus- or species-level identification.

All microscopic examinations were performed by laboratory personnel trained and experienced in veterinary parasitology. Samples were considered positive when at least one morphologically compatible strongyle-type egg was observed in the McMaster counting chamber. Therefore, all subsequent analyses and interpretations were based on positivity to strongyle-type gastrointestinal nematode eggs rather than on the identification of specific parasite genera or species.

Under the modified McMaster protocol used in this study, the multiplication factor of 50 corresponded to an analytical detection limit of 50 EPG. Therefore, the observation of a morphologically compatible strongylid-type egg in the counting chamber corresponded to a minimum detectable count of 50 EPG to be considered positive. Samples with egg burdens below this analytical threshold could potentially have been classified as negative, and this limitation should be considered when interpreting positivity estimates and low-intensity infections.

### 2.10. Data Collection and Study Variables

#### 2.10.1. Data Collection

Information was collected at both the animal and farm levels using a standardized data collection form specifically designed for this study. Animal-level information was obtained through direct observation, physical examination, and consultation of farm records, when available. Farm management information was obtained through a structured interview with the owner or the person responsible for herd management. The data collection form included variables related to animal identification, age, sex, and physiological state. Information was also recorded on the production system, stocking rate, deworming practices, pasture rotation, and the geographic location of each farm. Data on management-related variables were obtained directly from the producers during the interview.

#### 2.10.2. Dependent Variable

The primary dependent variable was positivity to strongyle-type gastrointestinal nematode eggs, classified as positive or negative according to the coproparasitological examination performed using the modified McMaster technique. An animal was considered positive when at least one morphologically compatible strongyle-type egg was observed during microscopic examination. Because eggs of trichostrongylid nematodes are morphologically indistinguishable, positivity referred exclusively to strongyle-type eggs and not to any specific parasite genus or species.

The secondary outcome variable was parasite burden, expressed as eggs per gram of feces (EPG), calculated using the modified McMaster technique with a multiplication factor of 50. EPG values were analyzed as a continuous quantitative variable and were subsequently categorized as mild (<500 EPG), moderate (500–1500 EPG), or severe (>1500 EPG) infection for descriptive purposes.

#### 2.10.3. Independent Variables

The explanatory variables evaluated in this study were classified into animal-level variables and farm-level management variables. Animal-level variables included age, sex, and physiological status. Farm-level variables included production system, deworming frequency, pasture rotation, and stocking density.

Age was determined from farm records when available and, when necessary, confirmed by dental examination. Animals were classified as lambs (<6 months), juveniles (6–12 months), and adults (>12 months). Sex was recorded as male or female.

Physiological status was established from farm records, owner information, and physical examination. Animals were classified according to their physiological status as suckling, growing, pregnant, or non-pregnant. Suckling animals were those that were still dependent on maternal milk, whereas growing animals corresponded to animals in the active growth stage. Adult females were classified as pregnant or non-pregnant according to their reproductive status at the time of sampling.

Production systems were classified as intensive, semi-intensive, or extensive according to grazing management and feeding practices. Deworming frequency was categorized as <1 time/year, 1–2 times/year, or >2 times/year based on information provided by the farmers. Pasture rotation was recorded as yes or no, and stocking density was classified as high or low–medium according to the number of sheep maintained per hectare under the management conditions of each production unit.

Stocking density was analyzed as a dichotomous farm-level variable using the operational threshold defined in Section 2.7: low–medium (<20 sheep/ha) and high (≥20 sheep/ha).

### 2.11. Statistical Analysis

Statistical analyses were performed using InfoStat 2020I/free. Descriptive analysis was performed using frequencies and percentages for categorical variables, while quantitative variables were summarized using mean and standard deviation or median and interquartile range, depending on their distribution. The positivity of the strongyle-type eggs was estimated with its respective 95% confidence intervals, and the parasite load was expressed as eggs per gram of feces (EPG). Associations between positivity and explanatory variables were initially evaluated using univariate logistic regression models with cluster-robust standard errors grouped by farm, estimating crude odds ratios (ORs) and their corresponding 95% confidence intervals.

Variables showing a *p*-value < 0.20 in the univariate analysis, along with variables considered biologically relevant according to previous epidemiological evidence, were entered into the initial multivariable logistic regression model. Variable selection followed a backward elimination procedure, assessing changes in regression coefficients and biological plausibility. Multicollinearity was assessed using the variance inflation factor (VIF), with values below 5 considered acceptable. Model calibration was evaluated using the Hosmer–Lemeshow goodness-of-fit test, while model discrimination was analyzed by calculating the area under the receiver operating characteristic (ROC) curve (AUC). Standardized residuals and Cook’s distance were examined to identify influential observations. To account for the hierarchical structure of the data, cluster-robust standard errors grouped by farm were used to correct the estimated standard errors for the non-independence of animals belonging to the same production unit.

As a sensitivity analysis, a mixed-effects logistic regression model was fitted using farm as a random intercept to explicitly account for the hierarchical structure of sheep nested within farms. The fixed-effect component included the variables retained in the final multivariable model. The estimates obtained from the mixed-effects model were compared with those from the primary logistic regression model with cluster-robust standard errors. Because only 14 farms were included, the variance of the random effect and model convergence were carefully evaluated, and the results were interpreted cautiously.

### 2.12. Ethical Approval and Animal Welfare

The study protocol was approved by the Bioethics Committee of the Vice-Rectorate for Research of the Universidad Estatal de Bolívar under approval code UEB-CEI-DIV-AU-2026-039. All animal-handling and sample-collection procedures were conducted in accordance with institutional biosafety and animal-welfare guidelines. We obtained informed consent from the owners or persons responsible for the participating farms before sample collection and questionnaire administration.

## 3. Results

### 3.1. Characteristics of the Study Population

A total of 70 sheep from 14 participating farms were evaluated. Juveniles represented 48.6% of the sampled animals, followed by adults (27.1%) and lambs (24.3%), and females accounted for 64.3% of the study population. Among the participating farms, extensive production systems predominated (50.0%), 71.4% reported deworming less than once per year, 64.3% did not implement pasture rotation, and 57.1% were classified as having high stocking density. Complete baseline characteristics are provided in Table S2.

### 3.2. Strongyle-Type Gastrointestinal Nematode Egg Positivity

Thirty-eight of the 70 evaluated sheep tested positive, corresponding to an overall positivity of 54.3% (38/70; 95% CI: 42.6–65.5%), whereas 45.7% (32/70) of the sampled sheep were negative. Positive animals were detected in all evaluated sectors represented by the participating farms.

### 3.3. Fecal Egg Counts and Parasite Burden

Among the 38 positive animals (Table 1), fecal egg counts ranged from 200 to 3500 EPG. The distribution of fecal egg counts was right-skewed, with a median of 785 EPG (IQR: 320–1452.5 EPG), whereas the arithmetic mean was approximately 1150 EPG (SD: 1056.1 EPG), indicating considerable variability in egg shedding among positive animals. Individual animal-level data are provided in Table S5.

**Table 1:** Distribution of fecal egg counts according to infection-intensity categories among sheep positive for strongyle-type gastrointestinal.

Infection Intensity	EPG Range	Number of Positive Sheep	Frequency (%)	Mean EPG
Mild	<500	16	42.1	300.6
Moderate	500–1500	13	34.2	1000.3
Severe	>1500	9	23.7	2875.0
Total	—	38	100.0	1149.7

EPG: Eggs per gram.

### 3.4. Factors Associated with Positivity

The highest proportion of positive animals was observed among juveniles (70.6%), followed by lambs (58.8%), whereas adults showed the lowest positivity (21.1%). Males had a higher proportion of positive animals than females (72.0% vs. 44.4%). According to physiological status, animals in the growing stage showed the highest positivity (61.5%), followed by suckling animals (58.8%) and non-pregnant ewes (44.4%), whereas no positive animals were detected among pregnant ewes. Regarding farm-level characteristics, higher positivity was observed among sheep from extensive production systems (61.5%), farms with deworming frequencies of less than once per year (56.9%), farms without pasture rotation (59.1%), and farms with high stocking density (59.1%) (Table 2).

### 3.5. Univariate Analysis

Table 3 presents the results of the univariate logistic regression analysis of the factors associated with positivity for strongyle-type gastrointestinal nematode eggs. Compared to adult animals, lambs (OR = 5.00; 95% CI: 1.76–14.22; *p* = 0.003) and juveniles (OR = 7.64; 95% CI: 2.30–25.33; *p* = 0.001) showed a higher probability of positivity. Likewise, males showed a higher probability of positivity than females (OR = 3.21; 95% CI: 1.30–7.95; *p* = 0.011). Regarding the production system, sheep raised in extensive systems showed a higher probability of testing positive than those kept in intensive systems (OR = 2.80; 95% CI: 1.71–4.58; *p* < 0.001), while the comparison between semi-intensive and intensive systems was not statistically significant (*p* = 0.077). No statistically significant associations were observed for deworming frequency, pasture rotation, or stocking density (*p* > 0.05). Variables with *p*-values < 0.20 in the univariate analysis were considered for inclusion in the multivariable logistic regression model.

### 3.6. Multivariable Logistic Regression

**Figure 2:**
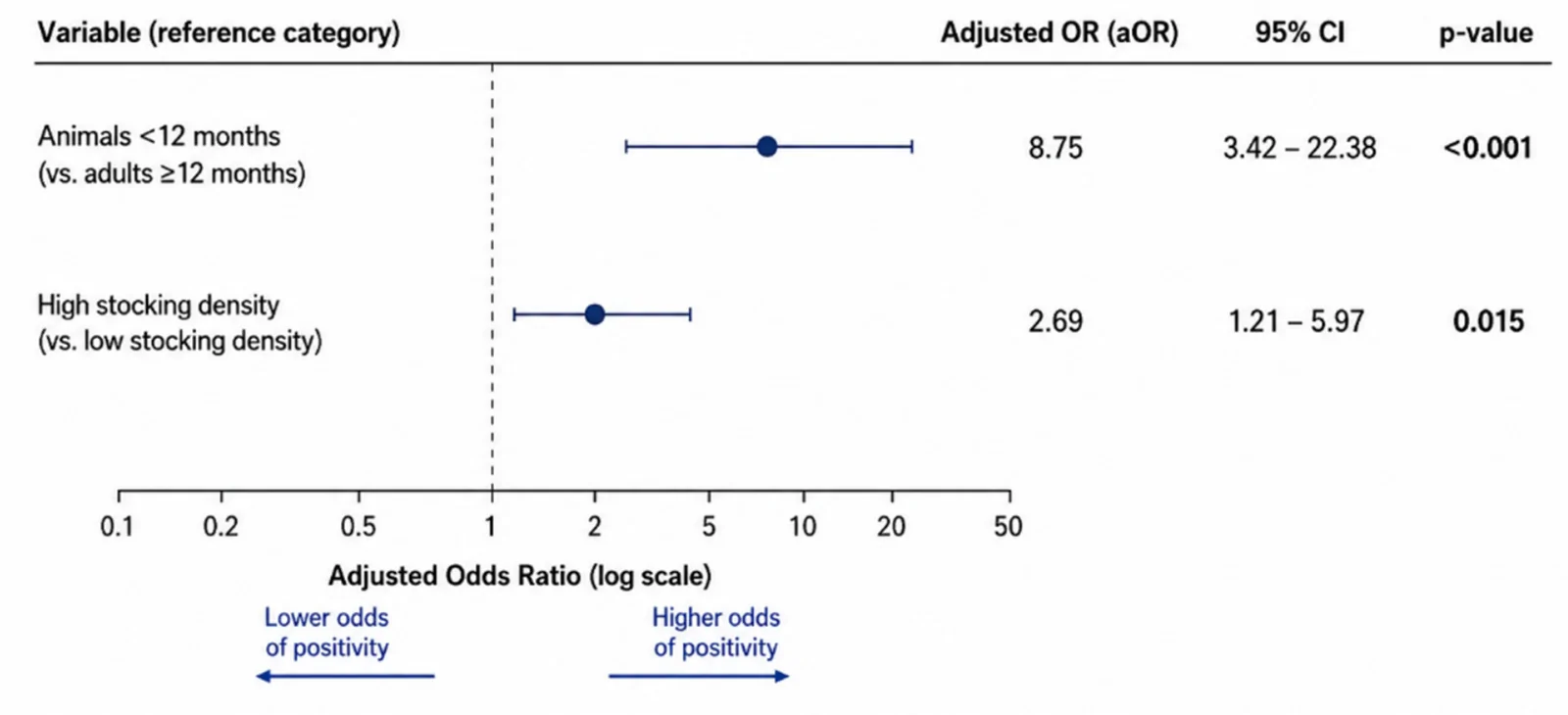
Forest plot of adjusted odds ratios (aORs) and 95% confidence intervals for factors associated with strongyle-type gastrointestinal nematode egg positivity in the primary multivariable logistic regression model. The vertical reference line at OR = 1 indicates the null value.

As shown in Figure 2, sheep younger than 12 months had the largest adjusted odds ratio (aOR = 8.75; 95% CI: 3.42–22.38), whereas high stocking density was also statistically associated with strongyle-type egg positivity in the primary multivariable analysis (aOR = 2.69; 95% CI: 1.21–5.97). Both confidence intervals remained above the null value (OR = 1). These estimates and their corresponding 95% confidence intervals are graphically presented.

As a sensitivity analysis, a mixed-effects logistic regression model was fitted with farm included as a random intercept. The estimated between-farm random-effect variance was approximately zero, indicating little detectable residual heterogeneity among farms after adjustment for the variables included in the model. In this analysis, sheep younger than 12 months remained significantly associated with positivity to strongyle-type gastrointestinal nematode eggs (OR = 8.75; 95% CI: 2.32–33.03; *p* = 0.001). High stocking density showed an effect estimate in the same direction as the primary analysis (OR = 2.69), but the confidence interval included the null value (95% CI: 0.89–8.14; *p* = 0.080) (Table 4). Therefore, the association between young age and positivity was robust across analytical approaches, whereas the association with stocking density was less stable.

**Table 4:** Sensitivity analysis using mixed-effects logistic regression with farm as a random intercept.

Predictor	Comparison	OR	95% CI	*p*-Value
Age	<12 months vs. adults	8.75	2.32–33.03	0.001
Stocking density	High vs. low	2.69	0.89–8.14	0.080

Note: Farm was included as a random intercept. The estimated random-effect variance approached zero, indicating limited detectable residual between-farm heterogeneity.

The final multivariable logistic regression model with cluster-robust standard errors grouped by farm showed that animal age and stocking density remained statistically associated with positivity to strongyle-type gastrointestinal nematode eggs after adjustment for the variables included in the model. Animals younger than 12 months exhibited significantly greater odds of positivity than adults (adjusted OR = 8.75; 95% CI: 3.42–22.38; *p* < 0.001). Likewise, sheep raised on farms with high stocking density had higher odds of positivity than those maintained under low–medium stocking density (adjusted OR = 2.69; 95% CI: 1.21–5.97; *p* = 0.015). These findings represent adjusted statistical associations and should be interpreted considering the cross-sectional design of the study and the clustered sampling structure.

Model diagnostics supported an adequate performance of the final multivariable model. The Hosmer–Lemeshow test showed no evidence of poor calibration (χ^2^ = 0.654; df = 2; *p* = 0.721), while discrimination was acceptable (AUC = 0.724; 95% CI: 0.630–0.811). No relevant multicollinearity was detected (maximum VIF = 1.03), and Cook’s distance did not identify highly influential observations (maximum = 0.071). Complete model diagnostic results are provided in Table S4.

## 4. Discussion

### 4.1. Positivity of Strongyle-Type Gastrointestinal Nematodes

The positivity rate observed in this study was 54.3%, indicating a considerable frequency of strongyle-type gastrointestinal nematode eggs among the sheep sampled on participating farms in the Guanujo parish. This value was lower than that reported in other sheep production systems in Latin America. Rojas-Hernández et al. [21] reported a positivity of 77.63% in grazing sheep in Mexico, while in Colombia a value of 83.2% of strongyle-type gastrointestinal nematodes was recorded [22]. Similarly, Martins et al. [23] reported a value of 77.0% of strongyle-type eggs in sheep in Brazil.

The differences between these studies and the results obtained in Guanujo should be interpreted with caution, as estimates can be affected by variations in sample design, climatic conditions, production systems, grazing pressure, deworming practices, and diagnostic methods used. For this reason, a direct comparison of percentages between regions does not necessarily reflect real differences in parasite transmission, but rather the specific methodological and production characteristics of each study.

In the Guanujo context, the observed positivity may be related to characteristics of the management systems evaluated, such as extensive grazing, animal density, and sanitary practices; however, these factors should only be interpreted as possible epidemiological explanations. Due to the cross-sectional design of the study, the results allow for the identification of statistical associations, but do not establish temporal or causal relationships between management conditions and strongyle egg positivity.

Furthermore, because parasitological identification was performed exclusively using the modified McMaster technique, the results do not allow the observed positivity to be attributed to specific genera such as *Haemonchus* or *Trichostrongylus*. Consequently, the 54.3% estimate should be interpreted as positivity for strongyle-type gastrointestinal eggs in the participating animals and farms, and not as a specific estimate of infection by a particular genus or as a positivity representative of the entire sheep population of the parish.

### 4.2. Parasite Burden

Among the 38 positive animals, fecal egg counts showed considerable variability, ranging from 200 to 3500 EPG, with a median of 785 EPG (IQR: 320–1453 EPG) and an arithmetic mean of approximately 1150 EPG. The higher mean relative to the median reflects the right-skewed distribution of fecal egg counts, indicating that a subset of animals exhibited substantially higher egg shedding. Comparable mean values have been reported in South America, including 1255 EPG in Colombia [22] and 1349.84 EPG in Brazil [23]. However, direct comparisons should be interpreted cautiously because differences in sampling design, diagnostic procedures, environmental conditions, and production systems may influence fecal egg counts.

The observed variability in EPG suggests heterogeneous parasite egg shedding among positive sheep and supports the use of the median and interquartile range, in addition to the arithmetic mean, to characterize parasite burden. Furthermore, because parasite identification in the present study was based exclusively on strongyle-type eggs, these fecal egg counts cannot be attributed to *Haemonchus*, *Trichostrongylus*, or any other specific gastrointestinal nematode genus. Therefore, the EPG values should be interpreted as an overall measure of strongyle-type gastrointestinal nematode egg shedding in the sampled animals rather than as genus- or species-specific parasite burdens.

### 4.3. Factors Associated with Strongyle-Type Gastrointestinal Nematode Egg Positivity

The multivariable analysis identified age and stocking density as the main factors associated with strongyle-type gastrointestinal nematode egg positivity. Young animals showed significantly higher odds of positivity than adults, and this association remained statistically significant in the mixed-effects sensitivity analysis, supporting the robustness of this finding. Similar epidemiological studies have reported higher frequencies of gastrointestinal nematode infection in young sheep, potentially related to differences in parasite exposure and the progressive development of acquired immune responses [13,24]. Nevertheless, because the present study is cross-sectional, this finding represents a statistical association and should not be interpreted as evidence of causality or inherent biological susceptibility.

High stocking density was also statistically associated with positivity in the primary multivariable analysis. However, although the magnitude and direction of this association remained similar in the mixed-effects sensitivity analysis, statistical significance was not maintained. Therefore, this result should be interpreted cautiously, particularly because stocking density was measured at the farm level and only 14 farms participated in the study. Previous studies have reported associations between grazing-related management conditions and gastrointestinal nematode infection, particularly in extensive systems and under environmental conditions favorable for parasite transmission [13,14,25]. In the present study, however, the production system itself did not remain independently associated with positivity after multivariable adjustment, suggesting that other correlated management characteristics may have partly explained its univariate association.

Similarly, sex, deworming frequency, and pasture rotation were not independently associated with strongyle-type egg positivity after multivariable adjustment. Their apparent associations in the univariate analysis may therefore reflect confounding or interrelationships among animal- and farm-level management variables. Although pasture rotation and strategic deworming are recognized components of integrated parasite-control programs [26], the present study did not demonstrate independent statistical associations between these practices and positivity. The relatively small number of farms, correlations among management practices, and the observational design may have limited the precision of these estimates. Overall, the findings support interpreting management variables collectively rather than as isolated determinants of strongyle-type gastrointestinal nematode egg positivity.

### 4.4. Implications for Parasite Control

Overall, the findings suggest that younger age was consistently associated with strongyle-type gastrointestinal nematode egg positivity, whereas high stocking density showed a less stable association across analytical approaches. Although integrated parasite control strategies, including appropriate anthelmintic use, pasture management, and stocking density control, are widely recommended to reduce parasite transmission, the present findings should be interpreted as statistical associations rather than evidence of causal relationships because of the cross-sectional design of the study.

## 5. Study Limitations

First, parasite diagnosis was based exclusively on the identification and quantification of strongyle-type eggs using coproparasitological techniques. Because neither larval culture nor molecular identification was performed, the parasite genera and species involved could not be determined. Consequently, the findings should be interpreted as representing positivity and fecal egg counts for strongyle-type gastrointestinal nematode eggs rather than infection by any specific genus or species. Future studies should incorporate larval culture and/or molecular methods, such as PCR-based identification, to improve taxonomic resolution and allow genus- or species-specific epidemiological interpretation.

Second, the cross-sectional design allowed the estimation of positivity and the evaluation of statistical associations between the investigated variables and strongyle-type egg positivity; however, it does not permit the establishment of temporal or causal relationships between potential exposure factors and infection. Furthermore, sampling was conducted during a single study period; therefore, seasonal variation in climatic conditions, pasture contamination, and parasite transmission dynamics could not be evaluated. Longitudinal studies incorporating repeated sampling across different seasons would provide a more comprehensive assessment of temporal infection patterns.

Third, the participating farms were selected by convenience according to accessibility and owners’ willingness to participate rather than through probabilistic sampling. Therefore, selection bias cannot be excluded, and the participating farms may not fully represent the diversity of sheep production systems in Guanujo parish. Consequently, the findings should primarily be considered applicable to the sampled animals and participating farms, and extrapolation to the entire sheep population of Guanujo parish or other production systems should be made with caution. Future studies should employ probabilistic or multistage sampling designs to improve farm representativeness and external validity.

Fourth, although the number of sampled animals was sufficient for the planned positivity estimation, the sample size was calculated using an absolute margin of error of 10%. This resulted in limited precision, as reflected by the relatively wide 95% confidence interval around the observed positivity estimate (54.3%; 95% CI: 42.6–65.5%). In addition, only 14 farms were included, which limited the precision and statistical power available for evaluating farm-level management variables. Future prevalence surveys should consider a smaller absolute margin of error, such as 5%, together with a larger number of animals and participating farms.

Finally, the hierarchical structure of the data should be considered when interpreting the associations observed in this study because individual sheep were clustered within 14 farms. Cluster-robust standard errors grouped by farm were used in the primary multivariable analysis to account for within-farm correlation. In addition, a mixed-effects logistic regression model with farm as a random intercept was fitted as a sensitivity analysis. The estimated between-farm random-effect variance approached zero, indicating limited detectable residual heterogeneity among farms after adjustment for the variables included in the model. Nevertheless, the relatively small number of farms limits the precision of farm-level and multilevel estimates. Additionally, although stocking density was quantitatively calculated as sheep per hectare, the threshold used to distinguish low–medium (<20 sheep/ha) from high (≥20 sheep/ha) stocking density was an operational cut-off based on the observed distribution of the participating farms rather than a previously validated epidemiological threshold. Therefore, the association observed for stocking density should be interpreted cautiously, particularly because this association was less stable in the mixed-effects sensitivity analysis. The applicability of the stocking-density threshold to other sheep production systems also requires further evaluation. Future studies including a larger number of farms and prespecified stocking-density thresholds are needed to provide more robust estimates of farm-level associations.

## 6. Conclusions

Strongyle-type gastrointestinal nematode egg positivity was detected in 54.3% of the sampled sheep. Animals younger than 12 months showed consistently higher odds of positivity across both analytical approaches. High stocking density was associated with positivity in the primary multivariable analysis, although this association was not statistically significant in the mixed-effects sensitivity analysis and should therefore be interpreted cautiously. These findings provide baseline epidemiological information for the participating sheep farms and support the consideration of animal age and farm-level management characteristics in parasite-control strategies.

## Figures and Tables

**Table 2:** Distribution of strongyle-type gastrointestinal nematode egg positivity according to animal- and farm-level characteristics.

Variable	Category	n	Positives	Positivity (%)
Age	Lambs (<6 months)	17	10	58.8
	Juvenile (6–12 months)	34	24	70.6
	Adults (>12 months)	19	4	21.1
Sex	Female	45	20	44.4
	Male	25	18	72.0
Physiological state	Suckling	17	10	58.8
	Growing	39	24	61.5
	Non-pregnant	9	4	44.4
	Pregnant	5	0	0.0
Production system	Extensive	39	24	61.5
	Semi-intensive	20	10	50.0
	Intensive	11	4	36.4
Deworming frequency	<1 time/year	51	29	56.9
	1–2 times/year	8	4	50.0
	>2 time/year	11	5	45.5
Pasture rotation	No	44	26	59.1
	Yes	26	12	46.2
Stocking density	High (≥20 sheep/ha)	44	26	59.1
	Low–medium (<20 sheep/ha)	26	12	46.2

**Table 3:** Univariate analysis of factors associated with positivity to strongyle-type gastrointestinal nematode eggs using logistic regression with robust standard errors grouped by farm.

Variable	Category	Reference	Crude OR	95% CI		*p*-Value
				Lower CI	Upper CI	
Age	Lamb	Adults	5.000	1.758–14.223		0.003
Age	Juvenile	Adults	7.636	2.302–25.329		0.001
Sex	Male	Female	3.214	1.304–7.925		0.011
System	Extensive	Intensive	2.800	1.713–4.578		0.000
System	Semi-intensive	Intensive	1.750	0.941–3.253		0.077
Deworming	<1 time/year	>2 times/year	1.582	0.641–3.902		0.319
Deworming	1–2 times/year	>2 times/year	1.200	0.551–2.614		0.646
Rotation	No	Yes	1.685	0.818–3.470		0.157
Density	High (≥20 sheep/ha)	Low–medium (<20 sheep/ha)	1.685	0.896–3.168		0.105

OR = Crude Odds Ratio; CI = 95% Confidence Interval. Variables with *p* < 0.20 in the univariate analysis, together with variables considered biologically relevant, were considered for inclusion in the multivariable model.

## Data Availability

The data presented in this study are available upon request to the corresponding author. The authors make available the information supporting the presented results and may provide it to researchers or interested parties upon reasonable request to the corresponding author, respecting applicable ethical and confidentiality principles.

## Supplementary Materials

The following supporting information can be downloaded at https://www.mdpi.com/article/10.3390/vetsci13090934/s1, Table S1: Distribution of sampled sheep among the production units included in the study in Guanujo parish, Ecuador; Table S2: Characteristics of the sheep and production units; Table S3: Flock size, effective grazing area, and calculated stocking density of the participating sheep farms; Table S4: Diagnosis of the multivariable logistic regression model; Table S5: Individual-level epidemiological characteristics, strongyle-type gastrointestinal nematode egg positivity, and fecal egg counts (EPG) of the 70 sheep included in the study.
