# Supplementary material for: Strongyle-Type Gastrointestinal Nematode Egg Positivity, Fecal Egg Counts, and Associated Factors in Sheep from Guanujo Parish, Ecuador
Source: Vet Sci. 2026 Sep 9;13(9):934. doi: 10.3390/vetsci13090934 (PMC13611951; doi:10.3390/vetsci13090934)
Supplement: Supplementary file 1 [file vetsci-13-00934-s001.zip › vetsci-4477541-supplementary.pdf]

Supplementary files

Table S1. Distribution of sampled sheep among the production units included in the study in Guanujo parish, Ecuador.

Sector	Farms code	Animals sampled
Centro de Guanujo	CGa	6
Centro de Guanujo	CGb	4
Nuevo Guanujo	NGa	4
Nuevo Guanujo	NGb	4
Carbón Chinipamba	CCh	5
Quinoa Corral	QCa	5
Quinoa Corral	QCb	4
Callanayacu	CAa	5
Callanayacu	CAb	4
Llullundongo	SjLa	8
Llullundongo	SjLb	4
Rayo Alto	RaAl	5
Rumipungo	RUa	6
Rumipungo	RUb	6
**Total**	**14**	**70**

Table S2. Characteristics of the sheep and production units

Variable<br>Animals (level n=70)	Category	n	%
**Age**	Lambs	17	24.3
	Juvenile	34	48.6
	Adults	19	27.1
**Sex**	Female	45	64.3
	Male	25	35.7
**Physiological status**	Growing	39	55.7
	Suckling	17	24.3
	Non-pregnant	9	12.9
	Pregnant	5	7.1
**Production units (n = 14)**			
**Production system**	Extensive	7	50.0
	Semi-intensive	5	35.7
	Intensive	2	14.3
**Deworming frequency**	<1 time/year	10	71.4
	1–2 times/year	2	14.3
	>2 times/year	2	14.3
**Pasture rotation**	Yes	5	35.7
	No	9	64.3
**Stocking density**	High	8	57.1
	Low–medium	6	42.9

Table S3. Flock size, effective grazing area, and calculated stocking density of the participating sheep farms.

Farm	Total sheep	Grazing area (ha)	Stocking density (sheep/ha)	Density category
CGa	10	0.3	33.33	High
CGb	15	0.4	37.50	High
NGa	15	1.2	12.50	Low–medium
NGb	12	1.0	12.00	Low–medium
CCh	16	2.0	8.00	Low–medium
QCa	10	0.5	20.00	High
QCb	12	1.5	8.00	Low–medium
CAa	12	1.0	12.00	Low–medium
CAb	12	0.5	24.00	High
SjLa	15	0.3	50.00	High
SjLb	10	1.0	10.00	Low–medium
RaAl	15	0.5	30.00	High
RUa	16	0.4	40.00	High
RUb	10	0.3	33.33	High

Table S4. Diagnosis of the multivariable logistic regression model

Indicator	Results
Number of animals	70
Number of farms	14
Positives animals	38
Hosmer–Lemeshow χ^2	0.654
Degrees of freedom	2
P-value	0.721
Area under the ROC curve (AUC)	0.724
95% CI of the AUC	0.630–0.811
Maximum Cook's distance	0.071
Influential observations	4
Maximum VIF	1.03

Table S5. Individual-level epidemiological characteristics, strongyle-type gastrointestinal nematode egg positivity, and fecal egg counts (EPG) of the 70 sheep included in the study.

Animal	Farm	Production system	Age	Sex	Physiological status	Stocking density	Positive	EPG
1	CGa	Extensive	Lamb	Female	Suckling	High	1	320
2	CGa	Extensive	Lamb	Male	Suckling	High	1	3045
3	CGa	Extensive	Adult	Female	Non-pregnant	High	1	510
4	CGa	Extensive	Lamb	Female	Suckling	High	1	250
5	CGa	Extensive	Juvenile	Male	Growing	High	0	—
6	CGa	Extensive	Juvenile	Female	Growing	High	0	—
7	CGb	Extensive	Lamb	Male	Suckling	High	0	—
8	CGb	Extensive	Lamb	Female	Suckling	High	1	600
9	CGb	Extensive	Lamb	Male	Suckling	High	1	200
10	CGb	Extensive	Juvenile	Female	Growing	High	1	280
11	NGa	Semi-intensive	Juvenile	Male	Growing	Low	1	2400
12	NGa	Semi-intensive	Lamb	Female	Suckling	Low	0	—
13	NGa	Semi-intensive	Lamb	Male	Suckling	Low	1	256
14	NGa	Semi-intensive	Juvenile	Female	Growing	Low	0	—
15	NGb	Semi-intensive	Juvenile	Male	Growing	Low	1	300
16	NGb	Semi-intensive	Lamb	Female	Suckling	Low	1	200
17	NGb	Semi-intensive	Lamb	Male	Suckling	Low	0	—
18	NGb	Semi-intensive	Lamb	Female	Suckling	Low	0	—
19	CCh	Extensive	Juvenile	Female	Growing	Low	1	1000
20	CCh	Extensive	Lamb	Male	Suckling	Low	1	350
21	CCh	Extensive	Adult	Female	Non-pregnant	Low	0	—
22	CCh	Extensive	Juvenile	Male	Growing	Low	1	2540
23	CCh	Extensive	Juvenile	Female	Growing	Low	0	—
24	QCa	Extensive	Juvenile	Male	Growing	High	1	430
25	QCa	Extensive	Adult	Female	Non-pregnant	High	0	—
26	QCa	Extensive	Lamb	Female	Suckling	High	0	—
27	QCa	Extensive	Juvenile	Female	Growing	High	1	1310
28	QCa	Extensive	Juvenile	Male	Growing	High	0	—
29	QCb	Semi-intensive	Juvenile	Female	Growing	Low	1	318
30	QCb	Semi-intensive	Adult	Female	Non-pregnant	Low	0	—
31	QCb	Semi-intensive	Lamb	Male	Suckling	Low	1	2100

32	QCb	Semi-intensive	Juvenile	Male	Growing	Low	0	—
33	CAa	Extensive	Juvenile	Female	Growing	Low	1	982
34	CAa	Extensive	Juvenile	Male	Growing	Low	1	1200
35	CAa	Extensive	Lamb	Female	Suckling	Low	0	—
36	CAa	Extensive	Adult	Female	Non-pregnant	Low	0	—
37	CAa	Extensive	Adult	Female	Pregnant	Low	0	—
38	CAb	Semi-intensive	Juvenile	Female	Growing	High	1	250
39	CAb	Semi-intensive	Lamb	Male	Suckling	High	1	320
40	CAb	Semi-intensive	Juvenile	Male	Growing	High	1	1500
41	CAb	Semi-intensive	Adult	Female	Pregnant	High	0	—
42	SjLa	Extensive	Juvenile	Female	Growing	High	1	3500
43	SjLa	Extensive	Juvenile	Male	Growing	High	1	650
44	SjLa	Extensive	Lamb	Female	Suckling	High	0	—
45	SjLa	Extensive	Juvenile	Female	Growing	High	1	2970
46	SjLa	Extensive	Adult	Female	Non-pregnant	High	1	385
47	SjLa	Extensive	Juvenile	Male	Growing	High	1	430
48	SjLa	Extensive	Juvenile	Female	Growing	High	1	3000
49	SjLa	Extensive	Adult	Female	Pregnant	High	0	—
50	SjLb	Semi-intensive	Juvenile	Male	Growing	Low	1	982
51	SjLb	Semi-intensive	Juvenile	Female	Growing	Low	0	—
52	SjLb	Semi-intensive	Juvenile	Male	Growing	Low	0	—
53	SjLb	Semi-intensive	Juvenile	Female	Growing	Low	0	—
54	RaAl	Intensive	Juvenile	Male	Growing	High	1	920
55	RaAl	Intensive	Juvenile	Female	Growing	High	1	320
56	RaAl	Intensive	Juvenile	Male	Growing	High	0	—
57	RaAl	Intensive	Adult	Female	Non-pregnant	High	0	—
58	RaAl	Intensive	Juvenile	Female	Growing	High	0	—
59	RUa	Extensive	Juvenile	Male	Growing	High	1	1200
60	RUa	Extensive	Juvenile	Female	Growing	High	1	1050
61	RUa	Extensive	Adult	Female	Non-pregnant	High	0	—
62	RUa	Extensive	Adult	Female	Non-pregnant	High	1	3320
63	RUa	Extensive	Juvenile	Male	Growing	High	1	3000
64	RUa	Extensive	Adult	Female	Pregnant	High	0	—
65	RUb	Intensive	Adult	Female	Non-pregnant	High	1	200
66	RUb	Intensive	Adult	Female	Pregnant	High	0	—
67	RUb	Intensive	Adult	Female	Non-pregnant	High	0	—
68	RUb	Intensive	Adult	Female	Pregnant	High	0	—
69	RUb	Intensive	Juvenile	Female	Growing	High	1	1100
70	RUb	Intensive	Adult	Female	Pregnant	High	0	—
